# Reacting, Retreating, Regulating, and Reconnecting: How Autistic Adults in the United Kingdom Use Time Alone for Well-Being

**DOI:** 10.1089/aut.2024.0148

**Published:** 2024-07-29

**Authors:** Florence Neville, Felicity Sedgewick, Stuart McClean, Jo White, Isabelle Bray

**Affiliations:** 1 Centre for Public Health and Wellbeing, University of the West of England, Bristol, UK.; 2 School of Education, University of Bristol, Bristol, UK.

**Keywords:** autistic well-being, neurodiversity, qualitative methods, social overwhelm, sensory overwhelm, self-regulating

## Abstract

**Background:**

Firsthand accounts by autistic people describe a need for regular time alone. However, there is little in the literature that explores (1) why time alone is desired, (2) how that time is spent, or (3) where that time is spent. This article describes a neurodiversity-informed, qualitative study that demonstrates the importance and purpose of “alone-time” for autistic adults.

**Methods:**

We interviewed 16 autistic adults living in the United Kingdom about how and where they spent their “alone-time” and the benefits experienced from this time. We conducted the interviews online, some using a video link, and some using a synchronously accessed text-based document, according to the participants’ preferences.

**Results:**

We used Reflexive Thematic Analysis with the interview data to generate four qualitative themes as follows: (1) reacting to social and sensory overwhelm; (2) retreating from social and sensory overwhelm; (3) regulating, recovering, and recharging; and (4) ready to reconnect with others.

**Conclusions:**

These themes highlight a need for balancing social activities and spaces with time and space alone and the benefits of creating or protecting spaces, which encourage recovery from overwhelm.

**Community Brief:**

**Why is this an important issue?**

Autistic people often talk and write about how they need regular time alone for their well-being, but there is very little in the academic literature on the importance of “alone-time” or what “alone-time” might look like.

**What was the purpose of this study?**

We wanted to explore why autistic people might want time alone, how autistic people like to spend time alone, and where autistic people like to spend time alone.

**What did the researchers do?**

We interviewed 16 autistic adults living in the United Kingdom about how and where they spent their alone-time and what they felt the benefits of alone-time were. Then we analyzed what was said in the interviews using Reflexive Thematic Analysis.

**What were the results of the study?**

Four themes help describe what the autistic adults talked about: (1) reacting to social and sensory overwhelm; (2) retreating from social and sensory overwhelm; (3) regulating, recovering, and recharging; and (4) ready to reconnect with others. These themes highlight how social and sensory environments can be overwhelming, how finding quiet spaces and/or engaging with intense interests can be helpful, and how socializing can be made less overwhelming.

**What do these findings add to what was already known?**

This study is the first to explore why autistic people might choose to spend time away from social spaces, how they choose to spend this time, and where they choose to spend this time. The findings support previous research on sensory and social overwhelm and on well-being strategies that autistic people use to support their own well-being.

**What are potential weaknesses in the study?**

All the participants lived in the United Kingdom, had access to the internet, and were able to give informed consent, and most of the participants were White and female. We do not know if the findings are true for autistic people who are not represented by this sample (e.g., people who are further marginalized by not living independently).

**How will these findings help autistic adults now or in the future?**

This study shows the importance for autistic adults in balancing social activities and spending time in social spaces, with alone-time. It also shows the benefits of finding/creating and protecting spaces that are not overwhelming for autistic adults. These findings may help autistic adults explain their need for alone-time, which is useful information for people who support, live with, and work alongside autistic adults.

## Introduction

Autism diagnostic criteria such as the *Diagnostic and Statistical Manual of Mental Disorders*, Fifth Edition (DSM-5) and *International Classification of Diseases*, 11th Revision (ICD-11) reference atypicalities in social and communication behaviors and the presence of restricted and repetitive behaviors.^1,2^ However, neurodiversity-informed academics and advocates argue that autistic people experience the world differently from people who are not autistic and that understanding the embodied autistic experience should be of primary importance in defining autism.^3–5^ A neurodiversity-informed perspective recognizes the dynamics of social power inequalities, privilege, and oppression of neurodivergent people and considers that diversity among minds is natural, healthy, and valuable.^6,7^ In line with this approach, it should be recognized that autistic people may have different needs from people who are not autistic and that approaches to support autistic well-being might best be guided by autistic people themselves.

Since Kanner and Asperger documented their observations on autistic children’s apparent social withdrawal behaviors and desire for aloneness,^8,9^ assumptions have been made about autistic people’s lack of social motivation.^10^ Such assumptions are often based on observations of what are commonly considered to be disordered social behaviors, including lack of eye contact, avoidance of verbal communication, and social withdrawal. However, this perceived lack of social motivation is contradicted in the accounts of autistic people^11^; apparent social withdrawal behaviors do not necessarily indicate a lack of social interest. Rather, many autistic people report loneliness, a desire for community, and improved well-being when engaging with other people, particularly other autistic people.^12–14^

Social withdrawal behaviors may be driven by the overwhelm that autistic people commonly report from social and sensory environments.^15–17^ Being flooded with stimulation above the rate at which it can be processed can result in fear, confusion, and loss of verbal processing.^18,19^ This state of overwhelm can be experienced as *meltdowns*, *shutdowns*, and/or *autistic burnout*.^20^ Meltdowns and shutdowns should be understood as crisis experiences that result in involuntary explosive or withdrawal behaviors and that contribute toward feelings of exhaustion, shame, and failure. Multiple factors contribute to meltdowns and shutdowns, including anxiety, sensory processing difficulties, and social demands.^4^ Similarly, autistic burnout, defined as lasting at least 3 months and characterized by chronic exhaustion, loss of skills, and reduced tolerance to stimuli,^21^ differentiates from mainstream descriptions of burnout in that social interaction, social masking (adapting autistic behaviors to fit social majority expectations), and sensory stressors are contributory factors.^22,23^

Research that seeks to better understand and describe embodied autistic experiences suggests that autistic people experience a need for periods of solitude to protect against, alleviate, and recover from meltdowns, shutdowns, and autistic burnout.^12,21–23^ However, there do not appear to be any studies describing what those periods of solitude might look like or identifying how autistic people seek out or create spaces where they can be alone. Such knowledge would be useful for those seeking to understand autistic experiences and support autistic well-being. To address this gap in the literature, this qualitative study sought to explore firsthand accounts of autistic adults’ use of solitude or “alone-time” and comprises the first phase of a larger study examining alone-time as a well-being strategy for autistic adults.

## Positionality and Community Involvement

I (F.N.) am an autistic researcher, and I undertook the study under the academic supervision of non-autistic autism and/or well-being researchers (I.B., F.S., S.M., and J.W.). I take a pragmatic approach to research through understanding and documenting autistic people’s experiences and translating the results into actionable insights. As an autistic researcher and thus a member of collective global autistic and neurodivergent communities, I have added insight into community priorities. My research is supported by a Community Advisory Group (CAG) whose members each have lived experience of being autistic, prior engagement with emancipatory autism research, and a personal relationship with the study’s aims.

## Methods

### Participants

Between June and September 2021, I interviewed 14 participants via an online Microsoft Teams video link and 2 participants via a shared online OneDrive document, which was accessed synchronously. Criteria for inclusion in the study were that participants needed to be (1) autistic (including self-identified, formally assessed, and clinically diagnosed); (2) aged 18 or older; and (3) living in the United Kingdom. Participants were sought via Twitter posts from F.N.’s own account and, as such, were purposively selected but with an element of snowball sampling because of retweets by other Twitter users. I emailed a participant information sheet, a research privacy notice, and a consent form to those who expressed interest. On return of the consent form, I asked the participants to choose between an online video-interview or a synchronous text-based chat at a time of their choosing. I also asked participants to provide basic demographic information (Table 1) and their own pseudonyms. I informed them that any quoted data would be identified by their pseudonym and age only.

**Table 1.** Participants’ Demographic Characteristics

Gender	
Female	11
Male	4
Non-binary	1
Ethnicity	
Black British	1
Mixed Heritage	1
White British	11
White Irish	1
White Polish	1
White Welsh	1
Age	
18–30	4
31–40	1
41–50	5
51–60	5
61–70	1

### Ethics

The study was granted ethical approval by the University of the West of England Bristol’s Faculty Research Ethics Committee (HAS 21.03.128). Participants completed an online consent form in advance of their interviews, and I asked them for additional verbal or written consent at multiple points during the interviews. In addition to usual ethical requirements, particular consideration was given to the Academic Autism Spectrum Partnership in Research and Education’s (AASPIRE) practice-based guidelines for including autistic adults in research^24^—in particular, avoiding coercion and exploitation while maximizing autonomy and inclusion, ensuring that the consent process was accessible, offering multiple modes of participation, and creating an accessible interview guide. I designed the participant information sheet, consent form, and research privacy notice to be clear and accessible, and they were reviewed by the CAG.

### Procedure

In line with AASPIRE recommendations, a pre-interview preparation sheet, designed with the CAG, detailed how the interview would run, provided an opportunity for the participants to reflect on the interview questions in advance, collected basic demographic information, and asked the participants what accessibility accommodations would or might be needed. This shared document also outlined what questions would be asked during the interview and provided space for participants to answer these briefly in writing. This meant that the interview could be grounded in information already provided by the participants and allowed for easier participant-recall in the interview. Together with both the interviewer (F.N.) and the interviewees being autistic, this pre-interview preparation facilitated open and honest discussions,^24,25^ with detailed and enriched storytelling giving context and color to answers.

Although I conducted the interviews after the Spring–Summer 2021 COVID-19 lockdown restrictions had been eased, many participants acknowledged and discussed the impact of COVID-19 on their well-being. All interviews were conducted by F.N. and ran between 30 and 60 minutes. The interviews were semi-structured, following an interview schedule (Appendix 1) but encouraging participants to elaborate when new points of interest came up or had been previously mentioned on the preinterview preparation sheet. The researcher–participant power dynamics inherent in research was reduced by understanding that the participant’s contribution was expertise related to their own experiences and understandings.^26^

### Data analysis

I (F.N.) transcribed all video interviews and tidied up text-based interview documents for ease of analysis and then analyzed the data using Braun and Clarke’s Reflexive Thematic Analysis (Reflexive TA), which treats the subjectivity of the researcher as a resource, acknowledging that the researcher and participants will impact on each other’s understandings during data collection and analysis.^27^ The six-phase recursive approach of Reflexive TA facilitated deep engagement with the dataset to capture both *semantic*, surface-level and *latent*, deeper, or conceptual meanings. Transcription and reading all the data through several times enabled familiarization of the data. Coding was both *deductive*, creating researcher- and theory-led codes because of my background as an autistic researcher working from a neurodiversity-led perspective, and *inductive*, identified by the data itself. While coding the datasets in the analysis software package NVivo, I continually redefined the codes and their descriptions to make sure that they were meaningful and helpful in relationship to the research question, and F.S. reviewed them. I developed four initial themes through grouping the codes into meaningful and equally weighted code clusters, and then I revisited both the codes and the initial themes to ensure that each theme had identifiable boundaries and enough evidence to support them. Once I had developed the theme definitions, I structured the theme names to enhance existing alliteration. Moving back and forth between phases, a key feature of Reflexive TA, served to deepen understandings and interpretations of the data.

### Findings

#### Theme 1: Reacting to social and sensory overwhelm

Participants found that difficult, intense, or extended periods of social input, certain sensory input, and social masking were physically overwhelming and associated with emotional distress. Juggling social and sensory input with social masking was felt to result in exhaustion, anxiety, irritability, physical discomfort, confusion, and difficulties with executive function. Trying to contain these strong feelings contributed toward further exhaustion.

I find myself becoming agitated and then frustrated if I don’t have the space to myself for a really long time. I also get angry … So, it’s mostly frustration and people starting to irritate me like it doesn’t matter what someone does, it’s just frustrating. Like everything is wrong and it’s just really tiring. (Cody, 20)

Social and sensory distractions made it difficult to think clearly or to focus on completing tasks. This caused frustration and feeling isolated from others not understanding or appreciating the effort taken to try and block out these distractions.

My brain is always working, always busy, always thinking, overthinking a lot of the time. And if it’s not my thoughts it's other people’s thoughts in the house or other conversations that are going on. And it’s just like I need there to be less sensory input reaching my brain because I’m dealing with everything that’s going on in my head and dealing with external input. It can become overwhelming. (Carys, 51)

It is very tiring and if it’s extreme, I can end up becoming mute. I can’t process any more enough to reply or ask questions or engage in any way. (Jae, 58)

Following social rules associated with non-autistic social behavior involved masking more authentically autistic social behaviors. Most of the participants described how the continual monitoring of performing emotional states through widely expected verbal and nonverbal communication styles, taking part in small talk, and avoiding stimming (self-soothing repetitive behaviors) also contributed toward exhaustion and feelings of overwhelm.

There are habits I only feel comfortable with showing when I’m by myself. I mean that I get self-conscious stimming in front of other people, but I feel overwhelmed when I have to consciously avoid stimming. (Tom, 18)

Overwhelm was often felt as a physical sensation. Some described a feeling of dangerous internal pressure threatening to explode or a “traffic jam of processing” (Emily, 27), and others described discomfort or even pain.

That sort of tunnel vision thing begins to happen and there’s tightness and the feeling that something is building inside and [I’ve] just got to do something with that because it’s just going to burst … it’s a clue that I need to stop. (Daisy, 47)

… that twitchy, all edges thing of—I don’t know what to do with myself, I need to do something with myself, I’ve gone sensory processing wrong, my skin feels wrong, everything feels a bit wrong, I’m all edges, I don’t know what to do. (Kirsty, 44)

Overwhelm reduced the energy for self-care and being able to recover easily from experiencing difficult or intense social and sensory environments.

Sometimes I get home and I’m like: I can’t think, I don’t know what I’m doing, I don’t know what stuff is. And it’s like—yeah you were around people too long. (Emily, 27)

#### Theme 2: Retreating from social and sensory overwhelm

It was important for the participants to have spaces they could retreat to, where they felt protected from negative sensory input, soothed by positive sensory input, and that marked clear protective boundaries from other people. These were generally rooms at home, with environments moderated to suit sensory needs, or in natural spaces where sensory input was felt to be positive.

A lockable bedroom, garage, or converted shed could become an important sanctuary and those who lived alone described the relief of locking their front door or turning their phones off to create barriers against the outside world.

The cats will come in with me so it’s a nice place [the shed] to enjoy the company of the cats. They really like the vibe in there, and I might just have the radio on there. I can hear the birds tweeting, the trees just waving, just having this great place to just go ahhh. (Lori, 53)

I’m very happy … just shutting the door. (John, 48)

Six key requirements, with varying levels of overlap, included the following: (1) clear, minimalist spaces; (2) spaces where the temperature, light, sounds, and smells could be controlled; (3) spaces that felt cozy; (4) visually interesting spaces; (5) spaces that felt safe and familiar; and (6) spaces that were set up to accommodate any special interests.

This entire room was white and my whole house was white … But when I painted this room I came in to see if it was dry. All of a sudden, I just like cried because I hadn’t realised that the white was stressing me out and I realised I was so calm … I just hadn’t realised how stressed it was making me. (Emily, 27)

I like being alone at home, which is lovely because it’s my space and I know where things are, I know what the noises are. One of the things I do to sort of take myself down a bit, I will do my noise identification, which is just—that’s the traffic, that’s the cats snoring, that’s the fridge having a chat to me, that’s the neighbours—and it’s that quiet and being able to know what everything is that is happening and being in the quiet that’s really important. (Kirsty, 44)

Being alone was also helpful in terms of not feeling inhibited, enjoying pleasurable sensory input, and being able to think clearly.

If I want to start jumping up and down and pointing at something and giggling, I can do it and I can just enjoy the moment in any way I like. (Sarah, 47)

I’ve always done things like, you know, gone off into the mountains with a tent, and I’ve spent a few days walking out in wide open spaces but by myself, actually enjoying getting away from people and by myself to … kind of recharge and gather your thoughts about things and return refreshed. (Bill, 47)

Some planned for daily or weekly access to time alone, by asking household members to leave the house for a while, by putting out visual cues asking not to be disturbed, or by going on errands to leave the house or a workspace for a bit. For several participants, waking up early in a busy household, or going to bed after everyone else, was the only way to ensure time alone, even though it meant missing out on sleep.

I get up super early so that I can have ideally three hours in the morning by myself. (Flavia, 57)

I used to sneak out and go on the swings at like 1 in the morning. I’d just sneak out the house and go across the street to the park and go on the swings. Cause I just needed like the dark and the rhythm of it and the quiet … And I would get up before everyone else and do whatever I needed to do and then by the time they were up I was in my room. But I was very sleep deprived. (Emily, 27)

#### Theme 3: Regulating, recovering, and recharging

Alone-time also gave opportunities for immersion through flow-state activities,^28^ focusing on details of interest and fictional worlds. These solitary activities, engaged in without the distraction of social input, were all considered to be helpful in processing thoughts and emotions that had previously built up, or conversely, to dissociate from them. Both processing and dissociating from thoughts and emotions through immersion had the effect of alleviating anxiety, worry, and perseverant thoughts.

It’s all solo activities, the kind of things that I can get really stuck into mental flow if I want to. And two or three hours can pass without me really realising. This just brings me joy. (Ceri, 29)

Most talked about fully immersing themselves in an intense interest such as making music, researching, gardening, or bike maintenance. These brought joy, and the chance to shift focus away from problematic distractions and triggers, in a way that one participant described as “therapeutic.” In this way, even activities that others might find stressful, such as commuting, gaming, and learning a new skill set, were felt to be relaxing.

It’s actually a nice feeling being in the flow state. Everything flows. It just feels nicer. No effort. I can feel the difference in my body. It’s smooth. It feels healthier. (Jae, 58)

So other people might think that sitting at a computer, you know doing research or whatever is not relaxing but to me it was yeah, well it is. I think it’s how my mind relaxes. (Susan, 61)

You just really have to focus on what you’re feeling [when motorbiking], like through your body, the feeling of the road and what you’re seeing and hearing around you and constantly processing that in an ongoing flow … And its relaxing … I don’t have to project an emotional state and I don’t have to think about things or worry about anything else other than the physics of it, like the traction of the road and the relative speed of other vehicles. (Jack, 40)

Several participants described the enjoyment of focusing on sensory details through photographing, sound recording, and writing.

I love taking photographs. And those photographs, and sort of slow-mos keep me going for ages afterwards. So, I take slow-mo shots of the sea. And audio of the sea coming and going. And the sound. Because it’s a very loud silence at the seaside. It just removes, it filters out the internal noises that we all have. (Maria, 53)

Flaky paint on doorhandles, and milk-bottles being put out and, just personal things that, even clothes on washing lines, which I probably shouldn’t have taken photos of, but building up a collection of that became a process of the rhythm of walking and the close observation [which has] the same effect on me as sitting and zoning out. (Daisy, 47)

Immersion in fictional worlds was enjoyable for many of the participants, and multiple readings or viewings were felt to relieve stress. These fictional worlds often felt safer, easier, and more comfortably predictable than real-world environments.

I could get the same amount of joy from reading something the 5th or 6th time as the 1st time around. Even though I can remember the phrases and the plot. You can lose yourself in the details, these are safe environments for you, even if they are unsafe, you know what’s happening and you can get absorbed in a world. (Ceri, 29)

Watching TV is a great way to distract myself after I’ve already been in a space that was overwhelming to me. TV helps me to avoid thinking about overwhelming and stressful situations for long enough that they stop making me feel as stressed out or overwhelming as they were before. (Tom, 18)

#### Theme 4: Ready to reconnect with others

Ultimately, spending time with others was desirable for most of the participants, particularly in small social groups, when having shared experiences, and/or when socializing with other neurodivergent people. However, without frequent access to alone-time in safe spaces or using flow-state activities, being sociable was a source of stress rather than enjoyment. It was important to feel rested and calm rather than going into a social situation with no energy reserves, and it was also important to know that there would be a chance to rest again afterward.

It’s not that I don’t like people, because I like people, to spend time with people. But only if I’m well enough and rested, prepared and have time after this. (Cody, 20)

When I was three months out walking in the countryside [alone], then I was much, much more able to deal with people. I was able to have conversations with people and feel a lot more relaxed. (Flavia, 57)

Shared interests could mean that socializing was based around a shared activity such as walking, making music, crafting, or watching films. This relieved the pressure to focus on masking through more non-autistic social behaviors and styles of communication.

If I watch TV with my family or go to the cinema with my friends, I have something other than them that I can focus on without being rude. I feel least overwhelmed spending time with people when we’re seeing things that I can get distracted by. (Tom, 18)

So, the reason that I go [to eco-therapy] is to get better at being around people again, and more confident in talking to people. Because there’s something we’re actually doing, you’ve got something to focus on and talk about. (Emily, 27)

We could have all three of us all sitting in the same room and we’re all sitting on our phones doing our own thing and that’s fine. (Carys, 51)

Some talked about how shared-interest activities were likely to attract other neurodivergent people, which made socializing easier. With other neurodivergent people, there was less pressure to adopt non-autistic social behaviors and communication and more understanding of sensory sensitivities, ultimately resulting in less social and sensory overwhelm.

Any friend I’ve felt a connection to over my life, any person I’ve felt easy with, has always turned out to be autistic or in some way neurodivergent. (Kirsty, 44)

## Discussion

This study is the first to specifically explore why autistic people might choose to spend time away from social spaces, how they choose to spend this time, and where they choose to spend this time. The findings offer a new perspective on the concept of alone-time as a well-being strategy. They also contribute toward a growing body of neurodiversity-informed autism research, which seeks to explore and redefine autistic well-being according to autistic people themselves.

Although the interviews took place after the Spring–Summer 2021 COVID-19 pandemic UK lockdowns, many of the participants described still being affected by mental health impacts mirrored in other research studies, including a reduction in social masking, greater control over the sensory environment, increased connection with family, and more time to engage in special interests as well as reduced social interactions and reduced opportunities to self-regulate through alone-time.^29,30,31^ Although each of these impacts are discussed here, the impact of lockdown was not specifically explored in the analysis as it was outside of the research scope and aims.

**FIG. 1.**
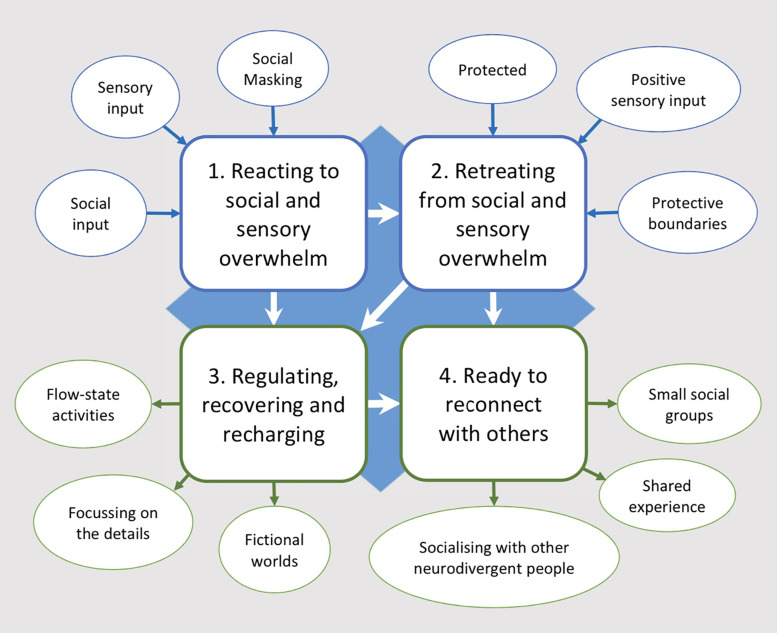
Thematic map showing (1) four numbered themes (in boxes), (2) 12 impacts to or from the themes (in ovals, with small arrows indicating direction of impact), and (3) the potential sequences between theme 1 and theme 4 (large arrows).

A thematic map (Fig. 1) shows two important aspects of the findings. First, the interactive relationships between each of the four themes (indicated by oblong outlines) suggest three potential pathways (indicated by large arrows) from *reacting to social and sensory overwhelm* to *ready to reconnect with others*. These pathways could be interpreted as a trio of linear recovery plans, which reduce the effects of overwhelm and resolve into feeling ready to reconnect with others, through self-managed strategies of retreating from social and sensory input and/or engaging with intense interests. Although *regulating, recovering, and recharging* through immersion activities were often talked about in isolation from *retreating from social and sensory distraction*, finding or creating “safe spaces” often appeared to provide a bridge between more extreme experiences of overwhelm or burnout and engaging with intense interests. Second, the impacts (indicated by oval outlines) to and from the four themes highlight how *reacting to social and sensory overwhelm* and *retreating from social and sensory overwhelm* could be seen as reactionary states, whereas *regulating, recovering, and recharging* and *ready to reconnect with others* could be seen as proactive and, thus, more empowering states.

Many elements of the thematic descriptions are recognized in the autism literature. For instance, the extrinsic factors considered to contribute toward overwhelm—social input, sensory input, and social masking—are all discussed in recent studies. Black et al.,^15^ Verhulst et al.,^16^ and MacLennan et al.^17^ discuss how social and sensory input correlates with anxiety and overwhelm for autistic people, and research into autistic social masking commonly lists anxiety and fatigue as consequences of behaving and communicating in non-autistic ways for the benefit of non-autistic people, including making eye contact, taking part in small talk, and refraining from stimming.^4,32,33,34^ Meanwhile, Raymaker et al.,^21^ Higgins et al.,^22^ Mantzalas et al.,^23^ and Quadt et al.^12^ discuss autistic adults’ needs and desires for spending time alone to recover from social and sensory overwhelm, something that these findings fully support.

In the current study, being able to retreat from social and sensory overwhelm required access to environments that offered a sense of being physically protected, positive sensory inputs, and clear protective boundaries from other people. Feeling physically safe, feeling comforted by the sensory environment, and having a sense of autonomy were applicable to both indoor and outdoor spaces and created a sense of sanctuary. The participants discussed highly individualized requirements from these environments, calling into question assumptions that highly minimalist rooms are universally calming for autistic people in distress. Practice recommendations should take heed of this inconsistency, for instance, the Care Quality Commission’s “Brief Guide to Seclusion Rooms” recommends that the design of seclusion rooms should include limited furnishings, externally controlled lighting, and externally controlled heating^35^; such environmental recommendations contrast strongly with the findings described in this article. If institutions are serious about addressing overwhelm and, thus, overwhelm behaviors, homes, educational, and care settings can, and should, provide “sanctuary” spaces that are designed with input from autistic people.

There is little research on outdoor environments that autistic people find soothing, although, in the current study, the experiences described from being in natural spaces mirror Brymer et al.’s research in the general population, which suggests that natural spaces provide “space for processing, a sanctuary from stressors and, at the same time acceptance and non-judgement.”^36^ Although such benefits are not unique to autistic people, the significantly higher rates of mental health challenges in the autistic population, such as addiction, anxiety, bipolar, depression, eating disorders, obsessive compulsive disorder, schizophrenia, self-harm, and suicidality, suggest that autistic people may have an increased need for sanctuary offered by natural spaces.^37–41^

Focusing on details, flow-state activities, and “losing oneself” in fictional worlds are sometimes seen as negative or symptomatic behaviors for autistic people.^42^ Yet, engaging with intense (also commonly referred to as “special” or “preferred”) interests is a positive and calming experience for autistic young people, leading to a reduction in anxiety; regaining control over negative thoughts, physical reactions, and emotions; and sleeping better,^20,43,44^ and autistic adults report that time spent on intense interests is also a recovery strategy for autistic burnout through sensory and emotion regulation and contributes toward long-term well-being.^22,23^ In the current study, all the participants discussed solitary activities and interests, and felt that engaging with these was beneficial to their well-being. Several participants also discussed how engaging with technology had similar benefits to activities not requiring technology. Although technology for recreation is often considered to have detrimental effects on well-being, recent studies with autistic young people show that engaging with technology such as online gaming is restful, joyful, fosters a sense of autonomy and belonging, and releases negative emotions.^45,46^

Autistic adults report high levels of loneliness,^12,47^ and several studies show that autistic adults gain a sense of well-being and belonging from feeling connected with and understood by other people.^13,49,50^ Many (but not all) participants in the current study expressed their enjoyment of the company of others and their struggles during lockdown without in-person socializing. They also talked about preferred social settings, particularly through shared activities, side-by-side activities, and meeting with small groups. Creating community through shared interests, experiences, and understandings appears to be easier with other autistic people, and socially identifying with the wider autistic community is reported to reduce depression symptoms, encourage a positive sense of identity, strengthen social connections, and raise collective feelings of self-esteem.^13,14,47,48,49^

This study’s findings support the social model of disability,^50^ in that some social and sensory environments were felt to be disabling and associated with high levels of distress, manifesting as physical discomfort, difficulties with communication, and a reduction in *executive functioning*, a collection of mental processes that include working memory, flexible thinking, and self-control. Equally, some environments were considered to support recovery, self-regulation, and/or social engagement. The understanding of disability as largely resulting from a poor fit between a person’s needs and the characteristics of an environment is consistent with a neurodiversity-informed perspective.^51^ This, in contrast to a pathology-informed perspective, considers that many difficulties experienced by autistic people are due to external factors rather than an impairment situated within an individual.^6,52^

### Limitations

At the time of recruitment and data collection, all the participants lived in the United Kingdom, had autonomy over their daily lives, and were able to provide written consent; it would be interesting to compare our findings with any future research that has similar aims but recruits autistic people with different background experiences and/or who experience marginalization from additional intersectional identities, such as skin color, culture, and being unable to live independently.

### Conclusions

Based on my (F.N.) lived experience and community knowledge, I aimed to explore why autistic people seek time alone, where that time might be spent, and what that time might look like. Spending time alone was considered by the participants to be a strategy that offered protection against and recovery from overwhelm, and although preferred environments and activities were variable, they offered sanctuary and/or opportunities for preferred self-regulation strategies. Understanding social and sensory overwhelm and the need for safe spaces and/or engagement with intense interests may help avoid certain crisis situations for autistic people or assist in shorter, easier, and less traumatic recovery time. Autistic adults learn for themselves how social and sensory input can be disabling and how attempts to “just push through” come at a cost, but these aspects are not always clear to autistic young people, or parents, carers, educators, or employers.

Using our findings to guide understanding and practice could benefit autistic people in many settings, including employment, education, or supported living. Working with autistic people of all ages to make and/or recommend simple environmental changes, reduce social communication pressures, recognize early signs of overwhelm, and engage with preferred sanctuary environments and activities as coping strategies would seem likely to reduce both crisis situations and long-term impacts.

As sensory and social overwhelm was a key finding in our study, future research should focus on the concept, risks, and protective factors of autistic overwhelm. In addition, large-scale quantitative studies would be useful for gaining generalizable results concerning how autistic adults might use alone-time to improve their well-being and whether alone-time benefits well-being.

## Dataset Statement

The dataset generated and analyzed for this research is not publicly available as consent was not provided by participants to share their data with third parties.

## Appendix

### Appendix 1: Interview Schedule (Semi-structured)

1. On your sheet you wrote that you do/don’t find it important to spend time alone. Can you tell me more about why this is?
2. Can you tell me more about the types of things you like to do when you are alone and what you find helpful about doing these things?
3. Can you tell me more about the spaces you like to be in and why you find these spaces helpful?
4. Can you tell me anything about how you find and create the spaces you like to be in alone and how you protect time to be alone in these spaces?
5. Can you tell me how it feels when you don’t get to spend time alone or when you can’t access the spaces you like to be in?
6. Can you tell me what you think the main benefits are, for you, of spending time alone in the spaces that suit you, and of doing the things you like to do alone?
7. Is there anything else that you’d like to tell me about?

## References

[1] American Psychiatric Association. *Diagnostic and Statistical Manual of Mental Disorders (DSM-5)* . American Psychiatric Association. 2013.

[2] World Health Organization . *International Statistical Classification of Diseases and Related Health Problems* (11th ed.). World Health Organization. 2019. https://icd.who.int/.

[3] Ratto A, Bascom J, daVanport S, et al. Centering the inner experience of autism: Development of the self-assessment of autistic traits. Autism Adulthood. 2023; 5(1):93–105; doi: 10.1089/aut.2021.0099 36941856 PMC10024271

[4] Welch C, Cameron D, Fitch M, Polatajko H. Living in autistic bodies: Bloggers discuss movement control and arousal regulation. Disabil Rehabil. 2021; 43(22):3159–3167; doi: 10.1080/09638288.2020.1729872 32097583

[5] Williams G . Perceptual deviants: understanding autistic subjectivities in a (not so) predictable world. In: *The Neurodiversity Reader: Exploring Concepts, Lived Experience and Implications for Practice* . ( Milton D , ed.) Pavilion Publishing and Media Ltd; 2020: pp. 35-40.

[6] Walker N . *Neuroqueer Heresies: Notes on the Neurodiversity Paradigm, Autistic Empowerment, and Postnormal Possibilities* . Autonomous Press, LLC; 2021.

[7] Walker N, Raymaker DM. Toward a neuroqueer future: An interview with Nick Walker. Autism Adulthood. 2021; 3(1):5–10; doi: 10.1089/aut.2020.29014.njw 36601271 PMC8992885

[8] Eisenberg L, Kanner L. Childhood Schizophrenia Symposium 1955. Early infantile autism 1943‐55. Am. J. Ophthalmol. 1956; 26(3):556–566; doi: 10.1111/j.1939-0025.1956.tb06202.x 13339939

[9] Asperger H . Die ‘Autistischen psychopathen’ im kindesalter. Eur Arch Psychiatry Clin Neurosci. 1944; 117(1):6–136; doi: 10.1007/BF01837709

[10] Chevallier C, Kohls G, Troiani V, Brodkin ES, Schultz RT. The social motivation theory of autism. Trends Cogn. Sci. 2012; 16(4):231–239; doi: 10.1016/j.tics.2012.02.007 22425667 PMC3329932

[11] Jaswal VK, Akhtar N. Being vs. appearing socially uninterested: Challenging assumptions about social motivation in autism. Behav Brain Sci. 2018; 42:e82–84; doi: 10.1017/S0140525X18001826 29914590

[12] Quadt L, Williams GL, Mulcahy JS, et al. “I’m trying to reach out, I’m trying to find my people”: Loneliness and loneliness distress in autistic adults. Autism Adulthood. 2023; doi: 10.1089/aut.2022.0062 PMC11447399 39371359

[13] Botha M, Dibb B, Frost DM. ‘It’s being a part of a grand tradition, a grand counter-culture which involves communities’: A qualitative investigation of autistic community connectedness. Autism. 2022; 26(8):2151–2164; doi: 10.1177/13623613221080248 35318862 PMC9597163

[14] Maitland CA, Rhodes S, O’Hare A, Stewart ME. Social identities and mental well-being in autistic adults. Autism. 2021; 25(6):1771–1783; doi: 10.1177/13623613211004328 34011188 PMC8323333

[15] Black MH, Clarke PJF, Deane E, et al. “That impending dread sort of feeling”: Experiences of social interaction from the perspectives of autistic adults. Res Autism Spectr Disord. 2023; 101(:102090; doi: 10.1016/j.rasd.2022.102090

[16] Verhulst I, MacLennan K, Haffey A, Tavassoli T. The perceived casual relations between sensory reactivity differences and anxiety symptoms in autistic adults. Autism Adulthood. 2022; 4(3):183–192; doi: 10.1089/aut.2022.0018 36606154 PMC9648696

[17] MacLennan K, O’Brien S, Tavassoli T. In Our Own Words: The complex sensory experiences of autistic adults. J Autism Dev Disord. 2022; 52(7):3061–3075; doi: 10.1007/s10803-021-05186-3 34255236 PMC9213348

[18] Bogdashina O . *Sensory Perceptual Issues in Autism and Asperger Syndrome: Different Sensory Experiences—Different Perceptual Worlds* . Jessica Kingsley; 2004.

[19] Murray D, Lesser M, Lawson W. Attention, monotropism and the diagnostic criteria for autism. Autism. 2005; 9(2):139–156; doi: 10.1177/1362361305051398 15857859

[20] Phung J, Penner M, Pirlot C, Welch C. What I Wish You Knew: Insights on burnout, inertia, meltdown, and shutdown from Autistic Youth. Front Psychol. 2021; 12:741421–741421. 34803822 10.3389/fpsyg.2021.741421 PMC8595127

[21] Raymaker DM, Teo AR, Steckler NA, et al. “Having All of Your Internal Resources exhausted beyond measure and being left with no clean-up crew”: defining autistic burnout. Autism Adulthood. 2020; 2(2):132–143; doi: 10.1089/aut.2019.0079 32851204 PMC7313636

[22] Higgins JM, Arnold SR, Weise J, Pellicano E, Trollor JN. Defining autistic burnout through experts by lived experience: Grounded Delphi method investigating #AutisticBurnout. Autism. 2021; 25(8):2356–2369; doi: 10.1177/13623613211019858 34088219

[23] Mantzalas J, Richdale AL, Adikari A, Lowe J, Dissanayake C. What Is Autistic Burnout? A thematic analysis of posts on two online platforms. Autism Adulthood. 2022; 4(1):52–65; doi: 10.1089/aut.2021.0021 36605565 PMC8992925

[24] Nicolaidis C, Raymaker D, Kapp SK, et al. The AASPIRE practice-based guidelines for the inclusion of autistic adults in research as co-researchers and study participants. Autism. 2019; 23(8):2007–2019; doi: 10.1177/1362361319830523 30939892 PMC6776684

[25] Pellicano E, Lawson W, Hall G, et al. “I Knew She’d Get It, and Get Me”: Participants’ Perspectives of a participatory autism research project. Autism Adulthood. 2022; 4(2):120–129; doi: 10.1089/aut.2021.0039 36605972 PMC9645671

[26] Karnieli-Miller O, Strier R, Pessach L. Power relations in qualitative research. Qual Health Res. 2009; 19(2):279–289; doi: 10.1177/1049732308329306 19150890

[27] Braun V, Clarke V. *Thematic Analysis, A Practical Guide* . SAGE; 2022.

[28] Csikszentmihalyi M . Learning, “flow,” and happiness. In: *Applications of Flow in Human Development and Education* . Springer: Dordrecht; 2014; doi: 10.1007/978-94-017-9094-9_7

[29] Pellicano E, Brett S, den Houting J, et al. COVID-19, social isolation and the mental health of autistic people and their families: A qualitative study. Autism. 2022; 26(4):914–927; doi: 10.1177/13623613211035936 34362263

[30] Bundy R, Mandy W, Crane L, et al. The impact of early stages of COVID-19 on the mental health of autistic adults in the United Kingdom: A longitudinal mixed-methods study. Autism. 2022; 26(7):1765–1782; doi: 10.1177/13623613211065543 35083922 PMC9483192

[31] Heyworth M, Brett S, den Houting J, et al. “I’m the Family Ringmaster and Juggler”: Autistic parents’ experiences of parenting during the COVID-19 pandemic. Autism Adulthood. 2022; 5(1):24–36; doi: 10.1089/aut.2021.0097 PMC10024268 36941857

[32] Bradley L, Shaw R, Baron-Cohen S, Cassidy S. Autistic adults’ experiences of camouflaging and its perceived impact on mental health. Autism Adulthood. 2021; 3(4):320–329; doi: 10.1089/aut.2020.0071 36601637 PMC8992917

[33] Cook J, Crane L, Bourne L, Hull L, Mandy W. Camouflaging in an everyday social context: An Interpersonal Recall Study. Autism. 2021; 25(5):1444–1456; doi: 10.1177/1362361321992641 33607921 PMC8264642

[34] Cummins C, Pellicano E, Crane L. Autistic adults’ views of their communication skills and needs. Int J Lang Commun Disord. 2020; 55(5):678–689; doi: 10.1111/1460-6984.12552 32618026

[35] Care Quality Commission. Brief Guide: Seclusion Rooms. 2019. Available from: https://www.cqc.org.uk/sites/default/files/Brief_guide_Seclusion_Rooms.pdf [Last accessed: 12December, 2022].

[36] Brymer E, Crabtree J, King R. Exploring perceptions of how nature recreation benefits mental wellbeing: A qualitative enquiry. Ann Leis Res. 2021; 24(3):394–413; doi: 10.1080/11745398.2020.1778494

[37] Fombonne E, Green Snyder L, Daniels A, Feliciano P, Chung W, The SPARK Consortium. Psychiatric and medical profiles of autistic adults in the SPARK Cohort. J Autism Dev Disord. 2020; 50(10):3679–3698; doi: 10.1007/s10803-020-04414-6 32096123

[38] Hand BN, Angell AM, Harris L, Arnstein Carpenter L. Prevalence of physical and mental health conditions in Medicare-enrolled, autistic older adults. Autism. 2020; 24(3):755–764; doi: 10.1177/1362361319890793 31773968 PMC7433648

[39] Nimmo-Smith V, Heuvelman H, Dalman C, et al. Anxiety disorders in adults with autism spectrum disorder: A Population-Based Study. J Autism Dev Disord. 2020; 50(1):308–318; doi: 10.1007/s10803-019-04234-3 31621020 PMC6946757

[40] Cassidy S, Bradley L, Shaw R, Baron-Cohen S. Risk markers for suicidality in autistic adults. Mol Autism. 2018; 9(1):42–42; doi: 10.1186/s13229-018-0226-4 30083306 PMC6069847

[41] Croen LA, Zerbo O, Qian Y, et al. The health status of adults on the autism spectrum. Autism. 2015; 19(7):814–823; doi: 10.1177/1362361315577517 25911091

[42] Ashinoff BK, Abu-Akel A. Hyperfocus: The forgotten frontier of attention. Psychol. Res. 2021; 85(1):1–19; doi: 10.1007/s00426-019-01245-8 31541305 PMC7851038

[43] Pavlopoulou G . A good night’s sleep: Learning about sleep from autistic adolescents’ personal accounts. Front Psychol. 2020; 11:583868; doi: 10.3389/fpsyg.2020.583868 33469436 PMC7814098

[44] Koenig PK, Williams HL. Characterization and utilization of preferred interests: A survey of adults on the autism spectrum. Occup Ther Ment Health. 2017; 33(2):129–140; doi: 10.1080/0164212X.2016.1248877

[45] Cheak-Zamora N, Odunleye O. Stress and coping in autistic young adults. Autism Adulthood. 2022; 4(3):193–202; doi: 10.1089/aut.2021.0043 36606158 PMC9645673

[46] Pavlopoulou G, Usher C, Pearson A. ‘I can actually do it without any help or someone watching over me all the time and giving me constant instruction’: Autistic adolescent boys’ perspectives on engagement in online video gaming. Br J Dev Psychol. 2022; 40(4):557–571; doi: 10.1111/bjdp.12424 35633283

[47] Milton D, Sims T. How is a sense of well-being and belonging constructed in the accounts of autistic adults? Disabil. Soc. 2016; 31(4):520–534; doi: 10.1080/09687599.2016.1186529

[48] Crompton CJ, Hallett S, Ropar D, Flynn E, Fletcher-Watson S. ‘I never realised everybody felt as happy as I do when I am around autistic people’: A thematic analysis of autistic adults’ relationships with autistic and neurotypical friends and family. Autism. 2020; 24(6):1438–1448; doi: 10.1177/1362361320908976 32148068 PMC7376620

[49] Cooper R, Cooper K, Russell AJ, Smith LGE. “I’m Proud to be a Little Bit Different”: The effects of autistic individuals’ perceptions of autism and autism social identity on their collective self-esteem. J Autism Dev Disord. 2021; 51(2):704–714; doi: 10.1007/s10803-020-04575-4 32607798 PMC7835309

[50] Oliver M . Theories of disability in health practice and research. BMJ. 1998; 317(7170):1446–1449; doi: 10.1136/bmj.317.7170.1446 9822407 PMC1114301

[51] Den Houting J . Neurodiversity: An insider’s perspective. Autism. 2019; 23(2):271–273; doi: 10.1177/1362361318820762 30556743

[52] Chapman R . Neurodiversity, disability, wellbeing. In: *Neurodiversity Studies: A New Critical Paradigm* . (Bertilsdotter Rosqvist H, Chown N, Stenning A, eds.) Routledge; 2020; pp. 57–72.

